# Endovascular treatment in ischemic stroke with active cancer: retrospective analysis of university stroke center data

**DOI:** 10.1186/s42466-025-00392-1

**Published:** 2025-05-19

**Authors:** Athina-Maria Aloizou, David-Dimitrios Chlorogiannis, Daniel Richter, Theodoros Mavridis, Dimitra Aloizou, Carsten Lukas, Ralf Gold, Christos Krogias

**Affiliations:** 1 Department of Neurology, St. Josef-Hospital, University Hospital of the Ruhr University, Bochum, Germany; 2 Department of Radiology, Brigham and Women’s Hospital, Harvard Medical School, Boston, MA USA; 3 Department of Neurology, Evangelisches Krankenhaus Herne, Academic Teaching Hospital of the Ruhr University Bochum, Bochum, Germany; 4 Department of Neurology, Tallaght University Hospital (TUH), The Adelaide and Meath Hospital, Incorporating the National Children’s Hospital (AMNCH), Dublin, Dublin, Ireland; 5 1st Neurology Department, Eginition Hospital, Medical School, National & Kapodistrian University of Athens, Athens, Greece; 6 Department of Nursing, National and Kapodistrian University of Athens, Athens, Greece; 7 Department of Neuroradiology, St. Josef-Hospital, University Hospital of the Ruhr University Bochum, Bochum, Germany; 8 Medical Faculty, Ruhr University Bochum, Bochum, Germany

**Keywords:** Ischemic stroke, Cancer, Coagulopathy, Thrombectomy, Endovascular treatment, Mortality

## Abstract

**Introduction:**

Active cancer (AC) associates strongly with ischemic stroke (IS). Intravenous thrombolysis (IVT) is often contraindicated in AC, and endovascular treatment (EVT) is considered the gold treatment standard, although data on its safety and efficacy is scarce.

**Methods:**

Digital records of patients receiving EVT in a tertiary university hospital with comprehensive stroke center from 2016 to 2022 were assessed. Demographic, clinical, and laboratory parameters were extracted and compared between patients with and without AC. In-hospital mortality was set as the primary outcome.

**Results:**

39 AC and 297 non-AC patients were included. No significant differences were reported in demographic and baseline stroke parameters (NIHSS, mRS, stroke etiology). In-hospital mortality did not differ between groups (11/39 vs. 57/297, *p* > 0.99). Successful recanalization, change in mRS and NIHSS from admission to discharge, periinterventional complications, and stroke-related mortality were also comparable. Significantly fewer AC patients received IVT. In the binary logistic regression analysis (adjusting for confounder variables), older age, large artery atherosclerosis, unsuccessful recanalization, and higher admission NIHSS were independent predictors of all-cause in-hospital mortality (aOR): 1.04, 95% confidence interval (CI): 1.01–1.08; OR: 3.21, 95% CI: 1.03–9.92, OR: 7.28, 95% CI: 3.61–15.1, OR: 1.07, 95% CI: 1.01–1.14, p-value < 0.05, respectively).

**Conclusions:**

EVT was shown as safe and effective in AC patients as in non-AC patients. Long-term functional outcomes are often poorer in AC, due to the cancer itself, but given how oncological treatment depends on functional status, AC patients should be considered for EVT.

## Introduction

An association of active cancer (AC) and acute ischemic stroke (AIS) has long been established [13], with several cancer-associated factors involved, namely hypercoagulability [11]. In this context, a new type of AIS has even been introduced; the “cancer-related stroke” (CRS) [7]. CRS pertains to an embolic AIS in a patient with AC and no other identifiable causes. Its characteristics include embolic strokes in various vascular territories, and evidence of hypercoagulability (e.g. elevated D-Dimers, recent history of venous thromboembolism) [3].

Regarding AIS treatment, intravenous thrombolysis (IVT) is administered at lower rates in AC patients, due to frequent contraindications, and clinician reluctance [25]. Meta-analyses reveal similar rates of intracranial hemorrhage (ICH), mortality, and functional outcomes between patients with and without AC undergoing IVT [14, 22, 23], so that cancer has now been mentioned in the American Heart Association guidelines [28], according to which IVT can be considered in AC patients with reasonable life expectancy. For endovascular treatment (EVT), several exclusion criteria of the landmark studies that established it [29] made the inclusion of AC patients a rarity [2, 6, 24]. Data regarding EVT in AC began to appear, through retrospective analyses after EVT establishment. A systematic review and a meta-analysis stated that EVT seems safe in AC, with comparable rates of hemorrhagic complications and successful recanalization, despite considerable inter-study heterogeneity [4, 5]. Mortality rates, often evaluated at 3 months, were shown elevated in AC, with cause of death often not relating to stroke, and outcomes were also inconsistently reported as poorer in AC [4, 5, 12]. Nevertheless, AC patients are yet to be mentioned in EVT guidelines, and definite conclusions regarding whether EVT should be applied in AC have not been drawn.

The study’s aim was to analyze data from a large tertiary University Hospital, regarding safety and outcome for AC patients treated with EVT for AIS. Select epidemiological and laboratory parameters were also compared between patients with and without AC.

## Methods

**Fig. 1:**
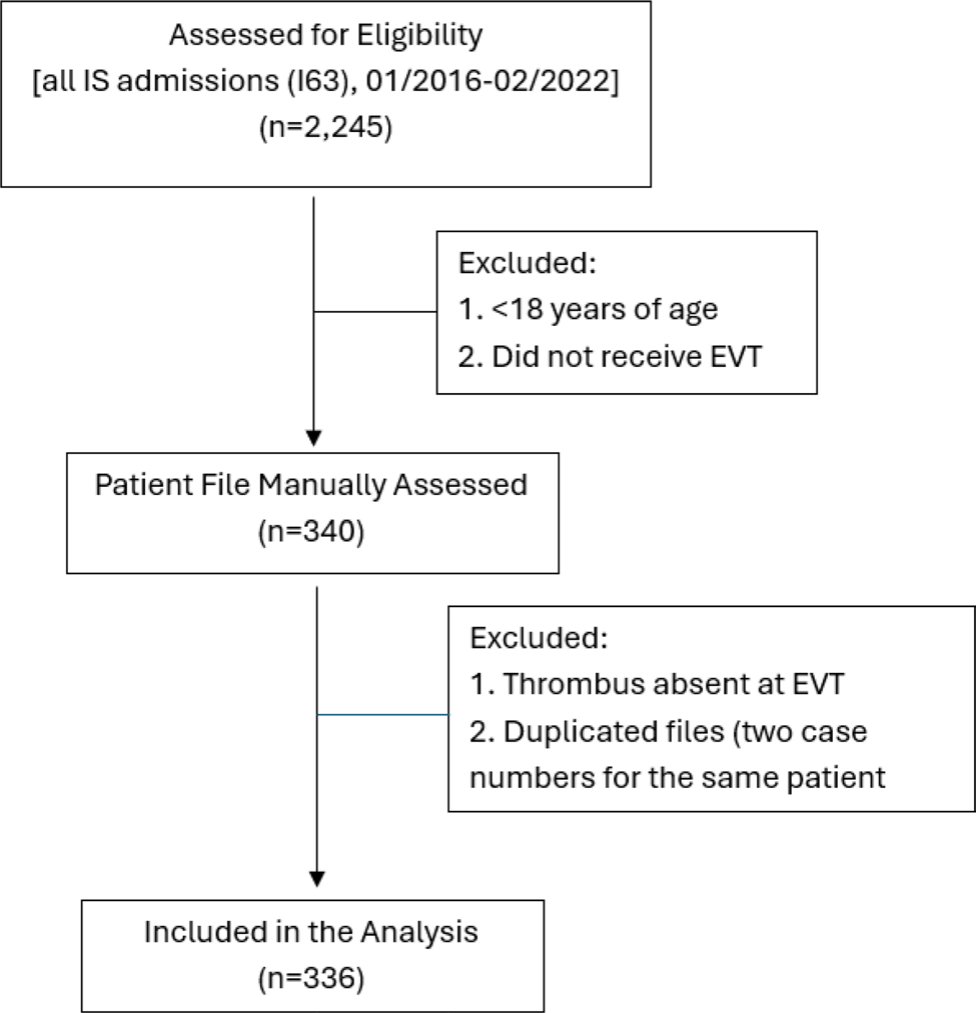
STROBE flowchart for patient inclusion selection

The digital records of all consecutive patients undergoing EVT at the St. Josef-Hospital Bochum, a university hospital of the Ruhr University of Bochum (RUB) in Germany, with a comprehensive stroke center, from January 2016 to February 2022 were individually assessed (STROBE flowchart [10], Fig. 1*)*. The study was approved by the RUB and no written informed consent was required (RUB Register Number 108 022 25352 7).

The following epidemiological data were extracted: age, sex, arterial hypertension, diabetes mellitus, history of previous stroke, prophylaxis at time of AIS (antiplatelet therapy or dual antiplatelet therapy (DAPT), Vitamin K Antagonists (VKA), low molecular weight heparins (LMWH) or direct oral anticoagulants (DOACs), or paused prophylaxis at time of AIS), presence of active or past cancer. Due to documentation discrepancies (quality management documentation vs. discharge letter and confirmed diagnoses, suspicion but no confirmation of diagnosis), the presence of atrial fibrillation could not be reliably extracted for every case and was thus omitted.

AC was defined as a malignant disease diagnosed or still being treated within one year of the AIS (before or after, for patients treated at our Institution’s oncological department), as stroke risk has been shown increased up until a year before cancer diagnosis [36], or known and not being treated (due to patient wishes or palliative care stage). Myelodysplastic syndromes, non-invasive skin cancers, and precancerous diseases were not considered as AC [34]. Previous cancer was considered as history of malignant disease curatively treated more than one year prior to the index AIS.

The following clinical parameters were extracted: occlusion of the anterior (ACi) or posterior circulation (PC), TOAST (Trial of Org 10172 in acute Stroke Treatment) classification [1], IVT administration, National Institute of Health Stroke Scale (NIHSS) and modified Rankin Scale (mRS) scores, ICHs, peri-interventional complications (artery dissections/wall hematomas, aneurysma spurium/hematoma at puncture location, occlusion of smaller arterial branches), in-hospital mortality (IHM). Patients were evaluated by the department’s neurologists upon admission and discharge. Deceased patients were awarded 42 points. The NIHSS and mRS score shift from admission to discharge, the number of patients with “excellent” and “good outcome” (mRS 0–1 and 0–2 respectively), and the rate of successful recanalization (Treatment in Cerebral Ischemia (TICI) Score ≥ 2b [17] were used as outcome metrics. Patients whose thrombus was no longer detectable at the time of EVT were excluded. TOAST classification was extracted from quality management documentation done through the treating neurologists and was thoroughly reevaluated according to patients’ records; cases with CRS suspicion, were homogeneously classified as of Unknown Etiology (UE). ICHs were classified according to the Heidelberg Bleeding Classification [35], and were further divided into a “hemorrhagic transformation” group for classes 1a/1b, “intraparenchymal hemorrhage within and beyond infarct with mass effect” for class 2, and “Other ICHs” for classes 3a-3d, besides the symptomatic/asymptomatic classification. Neurosurgical interventions (decompressive hemicraniectomy, trepanation, other operative treatment of ICH/cerebral edema) were also noted. Mortality was further classified as stroke-related and unrelated, while IHM was named the primary outcome. Stroke-related mortality included cases with malignant infarctions, large ICHs, prolonged reduced alertness, and aspiration as a result of the above, that led to death, with or without a palliative care setting being introduced. Unrelated mortality pertained to deaths due to reasons unrelated to stroke and its treatment, such as cardiac shock, septic shock not due to aspiration, pulmonary edema, and large intestinal bleeds in patients not receiving IVT.

Selected laboratory parameters were included: hemoglobin (Hb), hematocrit (HCT), platelets, international normalized ratio (INR), partial thromboplastin time (PTT), C-reactive protein (CRP), and lactate dehydrogenase (LDH).

For the statistical analysis, continuous variables were presented as mean and standard deviation (SD) if normality was followed, otherwise the median and interquartile range (IQR) were used. Categorical variables were presented as number (percentage). Comparisons between groups (AC and no AC) were performed using Fisher’s exact test when the expected counts were less than 5, and x^2^ if not, and Wilcoxon rank sum test. Univariate and multivariate logistic regression analyses were conducted to assess the influence of variables on overall survival. A stepwise backward elimination approach was employed. Variables were retained in the model if their removal caused a significant change in the fit, as determined by the Akaike Information Criterion. The confounders included in the model were AC, age, TOAST etiology, occlusion site, NIHSS score, Hb, CRP and LDH levels at admission, prophylaxis and IVT, which were selected a priori based on established biological associations or after univariate regression analysis with cutoff set at p-value < 0.2. A sensitivity analysis was performed by separately including only the a priori selected variables and those that reached univariate significance (*p* < 0.2). The results of these analyses were consistent with the primary analysis, confirming the robustness of the model. Significance for statistical difference was set at p-value < 0.05. The statistical computations were executed using R and R-Studio Version 4.3 (2024) [33].

## Results

In total, 336 patients were included, with 39 (11.6%) having AC. No significant differences were found in sex, age, hypertension, diabetes, history of previous stroke and cancer, and prophylaxis at admission (Table 1). AC patients demonstrated significantly lower Hb and HCT values and significantly higher CRP and LDH values (Table 1). Cancer type frequencies are presented in Table 2.

**Table 1:** Baseline demographic characteristics and laboratory values of EVT patients

	Overall *N* = 336	Active Cancer *N* = 39	Non-Active Cancer *N* = 297	*p*-value
**Sex**				0.49
Female	194 (58%)	25 (64%)	169 (57%)	
Male	142 (42%)	14 (36%)	128 (43%)	
Age*	75 (14)	73 (12)	76 (14)	0.10
**Hypertension**	273 (81%)	32 (82%)	241 (81%)	> 0.99
**Diabetes**	72 (21%)	11 (28%)	61 (21%)	0.30
**Stroke History**	63 (19%)	10 (26%)	53 (18%)	0.27
**Cancer History**	38 (11%)	8 (21%)	30 (10%)	0.062
**Prophylaxis at Admission**				0.065
Antiplatelet	96 (29%)	10 (26%)	86 (29%)	
VKA	8 (2.4%)	4 (10%)	4 (1.3%)	
LMWH	9 (2.7%)	1 (2.6%)	8 (2.7%)	
DOAC	32 (9.5%)	1 (2.6%)	31 (10%)	
Dual Treatment	11 (3.3%)	1 (2.6%)	10 (3.4%)	
None	147 (44%)	19 (49%)	128 (43%)	
Paused	14 (4.2%)	0 (0%)	14 (4.7%)	
Insufficient Treatment	19 (5.7%)	3 (7.7%)	16 (5.4%)	
HCT (%)*	38 (6)	33 (6)	39 (6)	< 0.001
Hb (mg/dL)*	12.87 (2.24)	10.97 (2.12)	13.11 (2.14)	< 0.001
CRP (mg/L)*	22 (34)	53 (52)	18 (29)	< 0.001
LDH (U/L)*	245 (109)	336 (214)	233 (80)	0.003
INR*	1.15 (0.26)	1.19 (0.28)	1.14 (0.26)	0.062
PTT (sec)*	36 (18)	36 (10)	36 (19)	0.74
Platelets (/µL)*	241,417 (84,297)	230,154 (114,044)	242,896 (79,709)	0.25

*mean (SD). Abbreviations: CRP: C-reactive Protein, DOAC: direct oral anticoagulants, Hb: hemoglobin, HCT: hematocrit, INR: international normalized ratio, LDH: lactate dehydrogenase, LMWH: low molecular weight heparins, PTT: partial thromboplastic time, VKA: Vitamin K Antagonists

**Table 2:** Active Cancer localization

**Cancer Localization**	
Lung Cancer	9 (23%)
Gastrointestinal Malignancy	8 (21%)
Breast Cancer	5 (13%)
Pancreatic Cancer	4 (10%)
Unknown Focus, metastatic	4 (10%)
Gastrointestinal & Lung Cancer	2 (5.1%)
Endometrial Cancer	2 (5.1%)
Mediastinal Tumor	1 (2.6%)
Cervical Cancer	1 (2.6%)
Ovarian Cancer	1 (2.6%)
Prostate Cancer	1 (2.6%)
Urinary Tract Cancer	1 (2.6%)

**Fig. 2:**
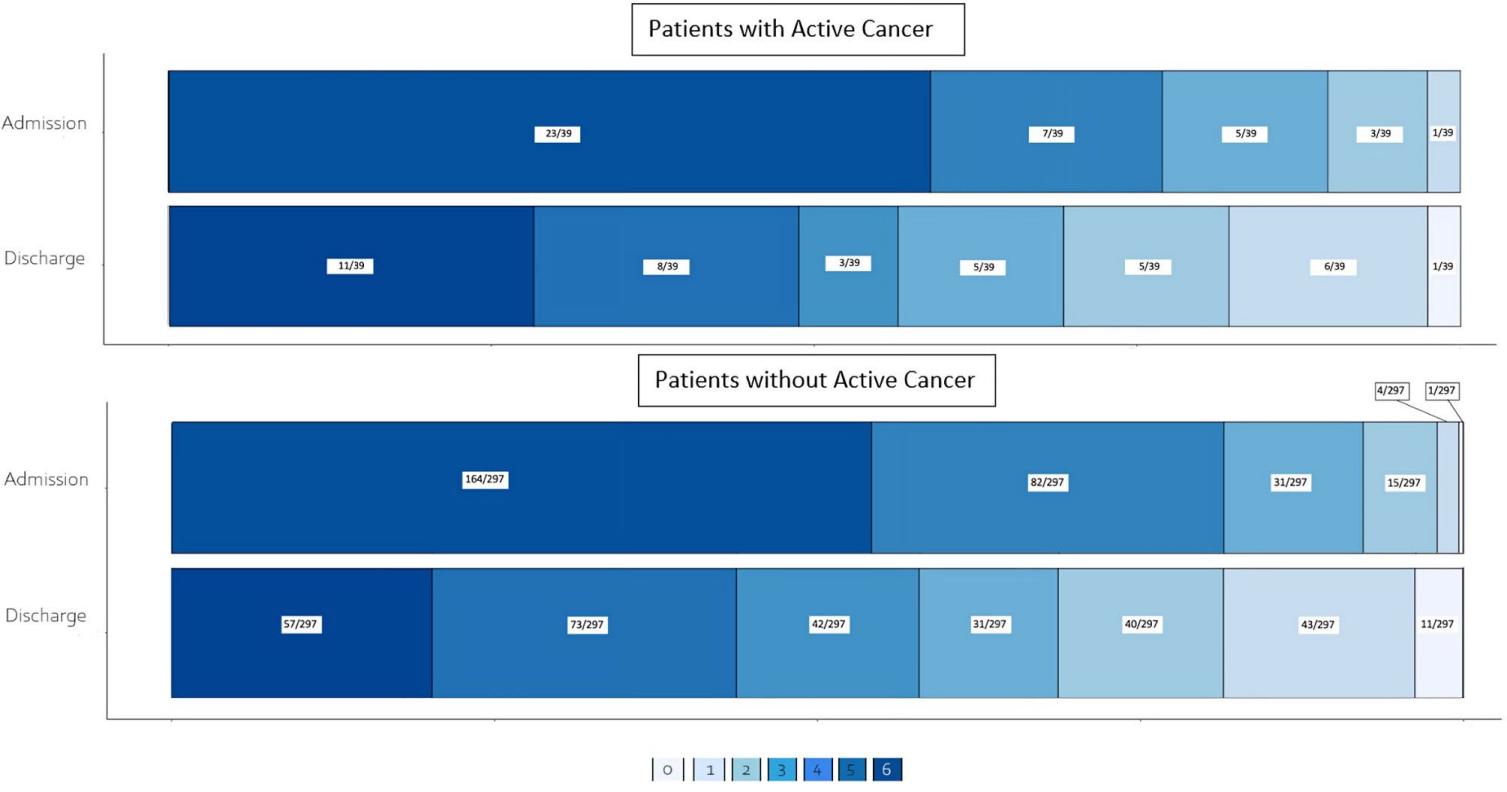
mRS Shift diagram for patients with and without active cancer

For AIS characteristics, EVT safety, and efficacy (Table 3), no significant differences were noted between AC and non-AC patients regarding TOAST classification, occlusion site, successful recanalization, NIHSS and mRS at admission and discharge, NIHSS/mRS shift, and excellent/good outcome (Fig. 2). IHM and stroke-related mortality, peri-interventional complications, ICH rates and types, and neurosurgical intervention rates were similar. The only significant difference was noted regarding IVT, with significantly fewer AC patients receiving IVT (15% vs. 39%, *p* = 0.004). Of the 6 AC patients receiving IVT, only one demonstrated a small hemorrhagic transformation with an otherwise very good recovery, and only one died. Four of the 6 had a good outcome. Of the 19 AC patients with UE, 14 fulfilled the criteria of CRS. For 2 EVT was unsuccessful, 5 deceased (4/5 deaths stroke related), and 4 of the remaining 9 did not improve after EVT. Regarding patients with intracranial tumors (*n* = 2), one patient in our cohort had a meningioma and no AC, and one had an intracerebral metastasis from their non-small cell lung cancer. None of these patients suffered an ICH or other periinterventional complications. The patient with the metastasis died, but due to reasons unrelated to stroke.

**Table 3:** Stroke characteristics, EVT safety and efficacy metrics

Items	Overall* N = 336	Active Cancer* N = 39	No Cancer* N = 297	*p*-value
**TOAST Category**				0.16
Cardioembolism	181 (54%)	16 (41%)	165 (56%)	
Large Artery Atherosclerosis	39 (12%)	4 (10%)	35 (12%)	
Unknown Etiology	110 (33%)	19 (49%)	91 (31%)	
Other Defined Etiology	6 (1.8%)	0 (0%)	6 (2.0%)	
**Occlusion Site**				0.42
Anterior Circulation	299 (89%)	33 (85%)	266 (90%)	
Posterior Circulation	35 (10%)	6 (15%)	29 (9.8%)	
Both	2 (0.6%)	0 (0%)	2 (0.7%)	
**Bridging IVT**	123 (37%)	6 (15%)	117 (39%)	**0.004**
**Successful Recanalization**	276 (82%)	32 (82%)	244 (82%)	> 0.99
**NIHSS at Admission**	13 (8, 18)	14 (7,18)	13 (8,18)	0.78
**NIHSS at Discharge**	9 (2, 22)	9 (2, 42)	9 (2, 20)	0.49
**mRS at Admission**	5 (4,5)	5 (4,5)	5 (4,5)	0.98
**mRS at Discharge**	5 (3,6)	5 (3,6)	5 (3,6)	0.54
**Change in NIHSS**	-3 (-9,7)	-2 (-8,18)	-3 (-9,6)	0.44
**mRS at Admission**				0.52
0	1 (0.3%)	0 (0%)	1 (0.3%)	
1	5 (1.5%)	1 (2.6%)	4 (1.3%)	
2	18 (5.4%)	3 (7.7%)	15 (5.1%)	
3	36 (11%)	5 (13%)	31 (10%)	
4	89 (26%)	7 (18%)	82 (28%)	
5	187 (56%)	23 (59%)	164 (55%)	
**mRS at Discharge**				0.82
0	12 (3.6%)	1 (2.6%)	11 (3.7%)	
1	49 (15%)	6 (15%)	43 (14%)	
2	45 (13%)	5 (13%)	40 (13%)	
3	36 (11%)	5 (13%)	31 (10%)	
4	45 (13%)	3 (7.7%)	42 (14%)	
5	81 (24%)	8 (21%)	73 (25%)	
6	68 (20%)	11 (28%)	57 (19%)	
**Change in mRS**	0 (-2,1)	0 (-2,1)	0 (-2,1)	0.48
**Excellent Outcome (mRS 0–1)**	61 (18%)	7 (18%)	54 (18%)	> 0.99
**Good Outcome (mRS 0–2)**	106 (32%)	12 (31%)	94 (32%)	> 0.99
**Mortality**	68 (20%)	11 (28%)	57 (19%)	> 0.99
**Stroke-Related Mortality**	51 (75%)	7 (64%)	44 (77%)	0.45
**Peri-interventional Complications**	36 (11%)	2 (5.1%)	34 (11%)	0.36
**Neurosurgical Intervention**	18 (5.4%)	2 (5.1%)	16 (5.4%)	0.70
ICH (all cases)**	85 (25%)	8 (21%)	77 (26%)	0.85
Hemorrhagic Transformation	31 (36%)	4 (50%)	27 (36%)	0.48
Intraparenchymal hemorrhage within and beyond infarct with mass effect	15 (18%)	0 (0%)	15 (20%)	0.66
Other Types of ICH	53 (63%)	4 (50%)	49 (64%)	0.48
Symptomatic ICH	19 (23%)	0 (0%)	19 (25%)	0.34

*n (%); Median (IQR). ** Some patients were allocated to two categories, for example when both hemorrhagic transformation and subarachnoid bleeding occurred. Abbreviations: ICH: intracranial hemorrhage, IVT: intravenous thrombolysis, mRS: modified Rankin Scale, NIHSS: National Institute of Health Stroke Scale, TOAST: Trial of Org 10,172 in Acute Stroke Treatment

In univariate analysis, predictors of all-cause IHM included older age, unsuccessful recanalization (TICI < 2b), Hb, CRP, LDH and higher NIHSS at admission (Table 4). In binary logistic regression analysis (adjusting for confounder variables), older age, large artery atherosclerosis (LAA), unsuccessful recanalization, and higher NIHSS at admission were independent predictors of all-cause IHM (adjusted odds ratio (aOR): 1.04, 95% confidence interval (CI): 1.01–1.08; aOR: 3.21, 95% CI: 1.03–9.92, aOR: 7.28, 95% CI: 3.61–15.1, OR: 1.07, 95% CI: 1.01–1.14, *p* < 0.05, respectively). AC was not associated with IHM (aOR: 0.95, 95% CI: 0.28–2.84, *p* > 0.9).

**Table 4:** Univariate and multivariate analysis for all-cause mortality

Characteristic	Univariate Analysis			Multivariate Analysis		
	OR	95% CI	*p*-value	OR	95% CI	*p*-value
**Sex**						
Male	1.19	0.69, 2.02	0.5	—	—	
**Active Cancer**						
Yes	1.65	0.75, 3.44	0.2	0.95	0.28, 2.84	> 0.9
**Age**	1.03	1.01, 1.06	**0.008**	1.04	1.01, 1.08	**0.01**
**Etiology (Baseline: Cardioembolic)**						
CRS	2.40	0.70, 7.42	0.14	2.86	0.44, 17.7	0.3
Large Artery Atherosclerosis	1.30	0.54, 2.90	0.5	3.21	1.03, 9.92	**0.042**
Unknown Etiology	1.14	0.61, 2.10	0.7	1.48	0.67, 3.28	0.3
Other	0.00		> 0.9	0.00		> 0.9
**Occlusion**						
Posterior Circulation	1.95	0.87, 4.12	0.09	1.16	0.42, 2.99	0.8
**Bridging IVT**	0.73	0.40, 1.27	0.3	1.01	0.49, 2.07	> 0.9
**Recanalization**						
Unsuccessful (< 2b)	6.26	3.41, 11.6	**< 0.001**	7.28	3.61, 15.1	**< 0.001**
**History of Cancer**						
Yes	1.06	0.43, 2.33	0.9	—	—	
**Hb**	0.86	0.76, 0.97	**0.015**	0.91	0.79, 1.05	0.2
**CRP**	1.01	1.00, 1.02	**0.003**	1.00	0.99, 1.01	0.4
**LDH**	1.00	1.00, 1.00	**0.026**	1.00	1.00, 1.00	0.6
**INR**	1.39	0.50, 3.51	0.5	—	—	
**PTT**	1.00	0.99, 1.02	0.6	—	—	
**Platelets**	1.00	1.00, 1.00	> 0.9	—	—	
**Hypertension**	1.00	1.00, 1.00	> 0.9	—	—	
**Diabetes**	1.16	0.60, 2.16	0.6	—	—	
**Prior stroke**	1.03	0.51, 1.98	> 0.9	—	—	
**Prophlylaxis at Admission**						
Antiplatelet	1.03	0.51, 1.98	> 0.9	1.03	0.51, 1.98	> 0.9
Dual Treatment	0.87	0.13, 3.59	0.9	1.05	0.14, 5.19	> 0.9
Insufficient	0.46	0.07, 1.72	0.3	0.54	0.07, 2.46	0.5
LMWH	1.11	0.16, 4.90	0.9	0.48	0.04, 3.47	0.5
DOAC	0.72	0.23, 1.90	0.5	0.51	0.13, 1.74	0.3
Paused	2.93	0.90, 9.06	0.063	1.56	0.34, 6.89	0.6
VKA	1.30	0.18, 5.98	0.8	1.66	0.20, 9.07	0.6
**NIHSS at Admission**	1.06	1.02, 1.10	**0.003**	1.07	1.01, 1.14	**0.019**
**mRS at Admission**	1.28	0.96, 1.77	0.12	0.83	0.52, 1.36	0.5

Abbreviations: CI: Confidence Interval, CRP: C-reactive Protein, CRS: Cancer-Related Stroke, DOAC: direct oral anticoagulants, Hb: hemoglobin, HCT: hematocrit, INR: international normalized ratio, IVT: intravenous thrombolysis, LDH: lactate dehydrogenase, LMWH: low molecular weight heparins, mRS: modified Rankin Scale, NIHSS: National Institute of Health Stroke Scale, OR: Odds Ratio, PTT: partial thromboplastic time, VKA: Vitamin K Antagonists

## Discussion

Our results show EVT as an equally safe and efficient modality for AIS in AC and non-AC patients. We revealed similar rates of all types of ICHs, peri-interventional complications, functional outcomes, and IHM between patients with and without AC. Additionally, AC was not a significant predictor of IHM.

Safety-wise, most studies also reported similar ICH rates after EVT for AC patients [8, 9, 20, 30–32, 34, 37]. Studies exploring CRS exclusively tended to report higher rates of hemorrhagic transformation and any ICH, though comparisons with control groups often lacked, and symptomatic ICHs were scarce [9, 15, 18]. Two meta-analyses also showed comparable rates for symptomatic ICH in AC and non-AC [5, 12]. No study reported higher rates of peri-interventional complications either [4, 32, 34, 37]. EVT was also similarly successful in the two groups, while NIHSS and mRS at discharge, and their shift, were comparable between groups, as shown in further studies too [16, 26, 31, 32, 34, 37].

Moving to IHM, some studies reported no differences [8, 21, 23, 27, 31, 37], and others revealed increased rates for AC, despite similar indices of ICH, other complications, and successful intervention [12, 30, 32, 34]. Interestingly, a large analysis of AIS hospitalizations reported an offset of the otherwise increased IHM for AC patients through recanalization treatment [27], highlighting EVT’s impact on overall prognosis of AIS in AC. We reported similar stroke-related and all-cause IHM rates in AC and non-AC. Most importantly, our regression analysis on IHM did not reveal AC and CRS as significant predictors of IHM either. This is reflected in studies with longer follow-ups, where 3-month mortality was often reported increased in AC, but the causes of death were more related to cancer than stroke [4, 5, 31]. Congruently, studies have shown higher mortality (IHM and up to 6 months) in CRS and advanced disease stages [25, 34, 37]. A similar analysis of a large database reported AC as an independent negative predictor of 3-month survival, with successful recanalization and pre-stroke independency being independent positive predictors; AC was not a significant predictor of functional status however [32], insinuating that AC affects survival and not the overall outcome after AIS treatment.

Concerning the other examined parameters, the comparable data on sex, age, hypertension, diabetes, history of AIS and cancer, stroke severity, occlusion localization, and TOAST classification, the percentage of AC patients and the reported cancer types, all reflected the relevant literature [18, 21, 26, 32]. The comparable TOAST distribution in AC and non-AC patients shows how all AC patients cannot placed in one big, homogeneous category, since traditional risk factors also play an important role in this patient subgroup as well. CRS is a relative rare entity, however suspicion should be raised, especially in patients where no other cause is identified.

Significant differences were identified in laboratory parameters (higher CRP and LDH, lower Hb and HCT), congruent with most available studies [18, 19, 37], and IVT, with AC patients receiving IVT less frequently than their non-AC counterparts, as shown in similar literature as well [16, 26, 27, 30]. Interestingly, only one of the 6 AC patients receiving IVT suffered a small, asymptomatic hemorrhagic transformation, with 4/6 presenting good outcomes; this reflects the ever-growing number of studies showing IVT’s safety in AC [23] and the inclusion of statements on AC patients in IVT guidelines [28].

Here, the study’s limitations need to be acknowledged. Firstly, the retrospective character of the study carries its inherent limitations; an attempt to countermeasure with regression analysis was made. Selection bias could also be discussed, since AC patients with very advanced disease stages may have not been subjected to any treatment, though the institution in general does not exclude such patients from EVT. Secondly, in the univariate analysis, AC and CRS due to the small patient numbers were included in the same model; a larger number of patients would have made our results even more robust and reliable. Thirdly, no follow-up data was available to assess long-term outcomes. Finally, as already mentioned, some parameters could not be reliably extracted for the whole cohort (e.g. exact presence of atrial fibrillation, pre-stroke functional status). Similarly, laboratory examinations linked to CRS, e.g. D-Dimers and fibrinogen, are not routinely examined and could not be included.

Conclusively, EVT for AIS appears to be safe and efficient in AC patients. The long-term outcomes in AC are often poorer, but since oncological treatments are heavily dependent on functional status, it is imperative to provide these patients with effective AIS therapies, in order to regain functionality and continue with oncological treatments.

## Data Availability

The datasets generated and/or analysed during the current study are not publicly available due to inclusion of patient data, but are available from the corresponding author on reasonable request.
